# Tomato drought-responsive transcription factor TINY1 suppresses embryonic growth

**DOI:** 10.1093/jxb/erag054

**Published:** 2026-02-06

**Authors:** Matar Azriel, Hagai Shohat, Dalia Blinderman, David Weiss, Yotam Zait

**Affiliations:** Institute of Plant Sciences and Genetics in Agriculture, The Hebrew University of Jerusalem, PO Box 12, Rehovot 76100, Israel; Institute of Plant Sciences and Genetics in Agriculture, The Hebrew University of Jerusalem, PO Box 12, Rehovot 76100, Israel; Institute of Plant Sciences and Genetics in Agriculture, The Hebrew University of Jerusalem, PO Box 12, Rehovot 76100, Israel; Institute of Plant Sciences and Genetics in Agriculture, The Hebrew University of Jerusalem, PO Box 12, Rehovot 76100, Israel; Institute of Plant Sciences and Genetics in Agriculture, The Hebrew University of Jerusalem, PO Box 12, Rehovot 76100, Israel; University of Sydney, Australia

**Keywords:** DREB, drought, embryo growth, gibberellin, TINY1, tomato, transpiration

## Abstract

Dehydration-responsive element-binding (DREB) transcription factors play an important role in plant responses to drought. DREB subfamily A4 contains a subgroup named TINY. Previous studies in Arabidopsis suggest that TINYs suppress plant growth and mediate abscisic acid (ABA)-induced stomatal closure. In this study, we investigated the function of the tomato drought-induced *TINY1*. Under drought conditions, the *tiny1* mutant lost turgor and wilted more rapidly than control M82 plants. However, this sensitivity was attributed to its larger leaf area, rather than intrinsic differences in drought response. Measurements of stomatal conductance, leaf temperature, and osmotic adjustment revealed no significant differences between *tiny1* and M82. Furthermore, whole-plant daily transpiration of M82 and *tiny1* with a similar leaf area showed no differences. Interestingly, the growth-promoting effect of *tiny1* was confined to early developmental stages; enhanced embryo growth and hypocotyl elongation, and accelerated emergence of the first true leaves—a trait that later contributed to increased leaf area. At later stages, the mutation had no observable impact on growth rate. Our results show increased gibberellin (GA) activity in the mature *tiny1* embryo and suggest that TINY1 suppresses embryonic growth by repressing GA biosynthesis through down-regulation of *GA 20-oxidase 4* (*GA20ox4*) gene expression.

## Teaser

The tomato drought-induced DREB transcription factor TINY1 suppressed embryonic and early seedling growth by inhibiting gibberellin activity, but it had no significant effect on drought response mechanisms.

## Introduction

Water deficiency directly and indirectly suppresses major biochemical pathways in plants, including photosynthesis and primary carbon metabolism (Tardieu *et al*., 2018). It also inhibits plant growth, flowering, and fruit development (Gupta *et al*., 2020). Plants use two major strategies to cope with drought: drought avoidance and drought tolerance (Skirycz and Inzé, 2010; Kooyers, 2015). ‘Drought avoidance’ is a major plant adaptation strategy to survive transient water-deficit conditions. To avoid drought stress, plants reduce their transpiration and can use the available water in the soil more slowly and for a longer period before the arrival of the next rain. Several mechanisms have evolved to reduce water loss under drought, including fast stomatal closure and long-term growth inhibition (Kalladan *et al*., 2017). Stomatal closure is regulated mainly by abscisic acid (ABA), which accumulates in response to drought and triggers a signaling cascade that leads to ion efflux, loss of guard cell turgor, and stomatal pore closure (Bharath *et al*., 2021). Growth inhibition is regulated by the reduced turgor pressure, which is the driving force for cell expansion (Ray *et al*., 1972; Ali *et al*., 2023), reduced levels of growth-promoting hormones, such as gibberellin (GA), and increased activity of ABA (Shohat *et al*., 2021). Tolerance to drought is acquired mainly through osmotic adjustment (Blum, 2017), scavenging of reactive oxygen species (ROS) (Noctor *et al*., 2014), activation of stress-related genes, and accumulation of drought-related proteins (Zhang *et al*., 2022).

The dehydration-responsive element-binding (DREB) family of transcription factors play a crucial role in plant responses to various abiotic and biotic stresses, including drought. These proteins belong to the APETALA2/Ethylene Responsive Factor (AP2/ERF) superfamily and are characterized by their ability to bind to specific DNA sequences called C-repeat (CRT)/dehydration-responsive elements (DREs) (Agarwal *et al*., 2006). DREBs affect and regulate various physiological and developmental stress responses, including stomatal closure, growth suppression, and induction of stress-related genes (Lata and Prasad, 2011; Sarkar *et al*., 2019). DREB proteins are divided into six subfamilies (A1–A6). Subfamily A4 contains 16 genes in Arabidopsis, including a subgroup named TINY (Nakano *et al*., 2006). Arabidopsis TINY1, a member of this family, is a unique transcription factor that connects both abiotic and biotic stress signaling pathways. It binds to both DRE and Ethylene-Responsive Element (ERE) sequences, making it a versatile regulator of stress responses in plants (Sun *et al*., 2008). TINY was first identified in an activation-tagging screen for gain-of-function mutations in Arabidopsis (Wilson *et al*., 1996). The mutant is dwarf due to a reduction in cell expansion and is therefore named TINY. *TINY* genes are induced by various abiotic stresses, including drought, cold, and salt. The triple Arabidopsis mutant *tiny1tiny2tiny3* has larger leaves and longer petioles (Xie *et al*., 2019), whereas TINY1-overexpressing plants have smaller leaves, increased drought-responsive gene expression, and they are hypersensitive to ABA-mediated stomatal closure (Sun *et al*., 2008; Xie *et al*., 2019). The effect of TINY on growth and drought response in Arabidopsis was associated with its interaction with the brassinosteroid (BR) pathway (Xie *et al*., 2019).

Tomato has six putative DREB-TINY genes, and drought conditions induced the expression of *TINY1* (Shohat *et al*., 2021). *SlDREB* (*TINY1* in Shohat *et al*., 2021) overexpression suppresses the expression of the gibberellin (GA) biosynthesis genes *ent-copalyl diphosphate synthase* (*CPS*), *GA 20-oxidase1* (*GA20ox1*), *GA20ox2*, and *GA20ox4*, reduces GA levels, inhibits growth, and promotes drought resistance (Li *et al*., 2012). Our previous study in tomato, using a *tiny1* CRISPR (clustered regularly interspaced palindromic repeats) mutant shows that the loss of TINY1 activity has no effect on *GA20ox1* and *GA20ox2* expression, but attenuated the induction of the GA-deactivating gene *GA2ox7* by drought (Shohat *et al*., 2021). Thus, the role of TINY1 in the regulation of GA accumulation under drought is not yet clear.

Here, we demonstrate that the loss of TINY1 activity in tomato promoted embryonic growth, hypocotyl elongation, and the formation of the first true leaf. However, beyond these early stages, *tiny1* did not influence the growth rate under either well-watered or drought conditions. Our results suggest that the loss of TINY1 increases GA activity in the growing embryo, probably due to the elevated expression of *GA20ox4*. Under drought conditions, *tiny1* mutants lost turgor more rapidly than the wild type (WT), but this was not attributable to delayed stomatal closure or increased stomatal conductance, but was due rather to the presence of an additional leaf which was produced early in seedling development but later resulted in a larger total leaf area.

## Materials and methods

### Plant materials and growth conditions

The loss-of-function CRISPR/Cas9 (CRISPR-associated protein 9)-derived *tiny1* alleles #1 (Shohat *et al*., 2021) and #23 and the transgenic line *35S:proΔ17* (Nir *et al*., 2017) are all in the M82 background. The *tiny1*#23 allele is a newly identified line not reported previously. This allele has an 89 bp deletion and an early stop codon after 270 bp (Supplementary Fig. S1). Plants were grown in a growth room set to a photoperiod of 12 h:12 h, day:night, light intensity of 150 μmol m^−2^ s^−1^, and room temperature of 25 °C. In other experiments, plants were grown in a greenhouse under natural day-length conditions, a light intensity of 700–1000 µmol m^−2^ s^−1^, and a temperature of 18–30 °C. Seeds were harvested from ripe fruits taken from plants grown in a greenhouse, thoroughly washed with water, and incubated overnight at 37 °C in 10% sucrose solution, then rinsed and surface-sterilized using 1% sodium hypochlorite followed by a wash with 1% Na_3_PO_4_·12H_2_O. Sterilized seeds were dried and stored at room temperature until use.

### Drought treatments

Plants were initially irrigated to saturation, after which irrigation was withheld to induce drought stress. Leaf relative water content (RWC; see below) was assessed.

### Measurements of relative water content

Leaf RWC of irrigated and drought-treated plants was measured as follows: FW was measured immediately after leaf detachment and then leaves were soaked for 24 h in 5 mM CaCl_2_ and the turgid weight (TW) was recorded. The leaves were subsequently dried at 55 °C for 72 h to obtain the DW. Leaf RWC was calculated using the following formula (Sade *et al*., 2009):


LeafRWC(%)=[(FW−DW)/(TW−DW)]×100.

### Stomatal conductance measurements

Stomatal conductance (*g*_s_) was measured on the terminal leaflet of the third fully expanded leaf (from the shoot apex) using an LI-COR Biosciences, Lincoln, NE, USA). All measurements were taken between 09.00 h and 11.00 h under controlled growth room conditions on 4-week-old plants.

### Thermal imaging

Thermal images were obtained using an A655sc, infrared camera with a 15° field of view (FLIR Systems, Wilsonville, OR, USA). The camera was mounted vertically above the plants to capture the entire canopy. Mean leaf temperature was calculated for the total leaf area using a customized region of interest (ROI) tool, following the manufacturer’s instructions.

### Leaf sap osmolality measurements

Terminal leaflets from the third fully expanded leaf (counting from the shoot apex) were collected, immediately frozen in liquid nitrogen, and stored at −80 °C. For sap extraction, samples were rapidly thawed at 37 °C and transferred into 1.5 ml Eppendorf tubes with perforated bottoms. These tubes were placed inside larger collection tubes and centrifuged at 12 000 *g* for 10 min to collect the sap. A 25 µl aliquot of the extracted sap was used to measure osmolyte concentration using a freezing-point depression (http://www.loeser-osmometer.de/home-eng.html) osmometer (Basic M, Löser Messtechnik, Germany).

### Whole-plant daily transpiration measurement

The whole-plant transpiration rate was measured using a high-throughput telemetric, gravimetric-based phenotyping system (Plantarray 3.0 system; Plant-DiTech, Israel), as described by Dalal *et al*. (2020). The experiment was conducted in the lysimeter greenhouse of the I-CORE Center for Functional Phenotyping at the Hebrew University (Rehovot, Israel) (http://departments.agri.huji.ac.il/plantscience/icore.phpon) under three irrigation regimes: control (full irrigation), moderate drought (irrigation at 50% of daily transpiration), and terminal drought (no irrigation). Plants were grown in 3.5 liter pots under semi-controlled environmental conditions with day/night temperatures of 30 °C/18 °C and exposed to the natural photoperiod and ambient light intensity (700–1000 µmol m^−2^ s^−1^). Each pot was placed on a load cell in a randomized block arrangement, and the soil surface was sealed to prevent evaporation. The data were analyzed using SPAC Analytics (Plant-Ditech, Yavne, Israel) software to obtain the following whole-plant physiological traits: daily transpiration, calculated as the weight loss between pre-dawn and sunset, and transpiration rate, calculated as the weight loss between two 3 min time points.

### Measurements of leaf area

Total leaf area per plant was measured using a LI- 3100 leaf area meter (LI-COR Biosciences).

### RNA extraction and cDNA synthesis

Total RNA was extracted from frozen plant tissues—including red ripe fruit seeds, hypocotyls, seedlings, or terminal leaflets of the third fully expanded leaf—using the RNeasy Plant Mini Kit (Qiagen, Hilden, Germany). For cDNA synthesis, 3 µg of total RNA was reverse transcribed using SuperScript II reverse transcriptase (18064014; Invitrogen, Waltham, MA, USA), following the manufacturer’s instructions.

### Reverse transcription–quantitative real-time PCR

Reverse transcription–quantitative real-time PCR (RT–qPCR) analysis was performed using an Absolute Blue qPCR SYBR Green ROX Mix (AB-4162/B) kit (Thermo Fisher Scientific, Waltham, MA, USA). Reactions were performed using a Rotor-Gene 6000 cycler (Corbett Research, Sydney, NSW, Australia). A standard curve was obtained using dilutions of the cDNA sample. Expression was quantified using ROTOR-GENE software (Corbett Research). Four independent technical repeats were performed for each sample. Relative expression was calculated by dividing the expression level of the examined gene by that of *ACTIN* and *TIP41* (Shohat *et al*., 2023). The gene to *ACTIN/TIP41* ratio was then averaged. The values for control treatments were set to 1. All primer sequences are presented in Supplementary Table S1.

### Library constructions and RNAseq analysis

Total RNA was extracted from terminal leaflets of the third fully expanded leaf using an RNeasy Plant Mini Kit (Qiagen). RNA-seq libraries were prepared at the Crown Genomics Institute of the Nancy and Stephen Grand Israel National Center for Personalized Medicine (INCPM), Weizmann Institute of Science, Israel, using the INCPM-mRNA-seq library preparation protocol. Briefly, the poly(A) fraction (mRNA) was purified from 500 ng of total input RNA, followed by fragmentation and synthesis of double-stranded cDNA. Libraries were purified using Agencourt AMPure XP beads (Beckman Coulter Life Sciences, Indianapolis, IN, USA), and subjected to end repair, A-tailing, adapter ligation, and PCR amplification. Library quality and concentration were assessed using a Qubit Fluorometer (Thermo Fisher Scientific) and a TapeStation system (Agilent Technologies, Santa Clara, CA, USA). Sequencing was performed on an Illumina NextSeq platform (Illumina, San Diego, CA, USA) using a 75 cycle high output kit, generating 75 bp single-end reads. Approximately 39 million reads were obtained per sample.

### Sequence data analysis

Poly(A/T) stretches and Illumina adapters were trimmed from the reads using cutadapt (Martin, 2011); resulting reads shorter than 30 bp were discarded. Reads were mapped to the reference genome *Solanum lycopersicum* release SL4.0 using STAR (Dobin *et al*., 2013) (with End to End option and out Filter Mismatch Nover Lmax set to 0.04). The annotation file was downloaded from SolGenomics, ITAG release 4.0. Reads with the same UMI were removed using the PICARD MarkDuplicate tool using the BARCODE_TAG parameter. Expression levels for each gene were quantified using htseq-count (Anders *et al*., 2015), using the GTF (Gene Transfer Format) file downloaded from SolGenomics. Differential expression analysis was performed using DESeq2 (Love *et al*., 2014) with the betaPrior, cooksCutoff, and independent filtering parameters set to False. Raw *P*-values were adjusted for multiple testing using the procedure of Benjamini and Hochberg (1995). The pipeline was run using snakemake (Köster and Rahmann, 2012).

### Microscopy and image analysis

#### Hypocotyl imaging

Hypocotyl tissues from seedlings were divided into three regions: upper, middle, and bottom. Hand-cut cross-sections from each region were mounted vertically on metal stubs coated with carbon tape. Imaging was performed using a Phenom ProX Scanning Electron Microscope (Thermo Fisher Scientific) equipped with a Backscatter Electron Detector (BSD). Images were acquired and processed using Phenom ProSuite software. Epidermal cell length was measured using ImageJ software (https://imagej.net/ij/). For each genotype, the final reported value represented the average epidermal cell length across the upper, middle, and bottom regions.

#### Embryo imaging

To isolate embryos, tomato seeds were imbibed in water for several hours and then dissected using a surgical blade and fine tweezers under a binocular microscope (Olympus, Waltham, MA, USA). Rescued embryos were imaged using a Nikon SMZ1270 stereo microscope equipped with a Nikon DS-Ri2 camera and NIS-Elements software (Nikon Instruments, Melville, NY, USA). Embryo and cotyledon lengths were measured using ImageJ software.

#### Hypocotyl diameter imaging (light microscopy)

Hand-cut hypocotyl cross-sections from seedlings were captured using a Leica DM500 microscope equipped with a Leica ICC50 W camera and Leica Application Suite software (Leica Microsystems, Heerbrugg, Switzerland). Hypocotyl diameters were analyzed using ImageJ software.

### Statistical analysis

All assays were conducted with three or more biological replicates and analyzed using JMP software (SAS Institute, Cary, NC, USA). Means comparison was conducted using ANOVA followed by post-hoc tests. For multiple comparisons among all groups, the Tukey–Kramer honest significant difference (HSD) test was used. For comparisons of experimental groups specifically against control, Dunnett’s test was applied. For single comparisons between two groups, Student’s *t*-test was used. A significance threshold of *P*<0.05 was applied for all tests.

### Gene annotation and accession numbers

Sequence data from this article can be found in the Sol Genomics Network (https://solgenomics.net/) under the following accession numbers: *ACTIN*, *Solyc11g005330*; *TIP41*, *Solyc10g049850*; *GA2ox1*, *Solyc05g053340*; *GA2ox2*, *Solyc07g056670*; *GA2ox4*, *Solyc07g061720*; *GA2ox7*, *Solyc02g080120*, *GA20ox1*, *Solyc03g006880*; *GA20ox2*, *Solyc06g035530*; *GA20ox3*, *Solyc11g072310*; *GA20ox4*, *Solyc01g093980*; *GA3ox1*, *Solyc06g066820*; *GA3ox2*, *Solyc03g119910*; *GA3ox3*, *Solyc01g058250*; *GA3ox4*, *Solyc05g052740*; *GA3ox5*, *Solyc00g007180*; *GID1b1*, *Solyc09g074270*; *TINY1*, *Solyc06g066540*; *TINY2*, *Solyc03g120840*; *TINY3*, *Solyc12g044390*; *TINY4*, *Solyc12g008350*; *TINY5*, *Solyc08g066660*; *TINY6*, *Solyc01g090560*; *Lectin-domain receptor-like kinas*, *Solyc07g055690*; *SEC3A*, *Solyc07g025170*; *SIP5CS1*, *Solyc06g019170*; *RD29A*, *Solyc03g025810*; *RD29B*, *Solyc01g009660*; *SlRAB18*, *Solyc02g084850*; *PROCERA*, *Solyc11g011260*.

## Results

### The loss of TINY1 increased the rate of water loss under drought conditions

**Fig. 1.**
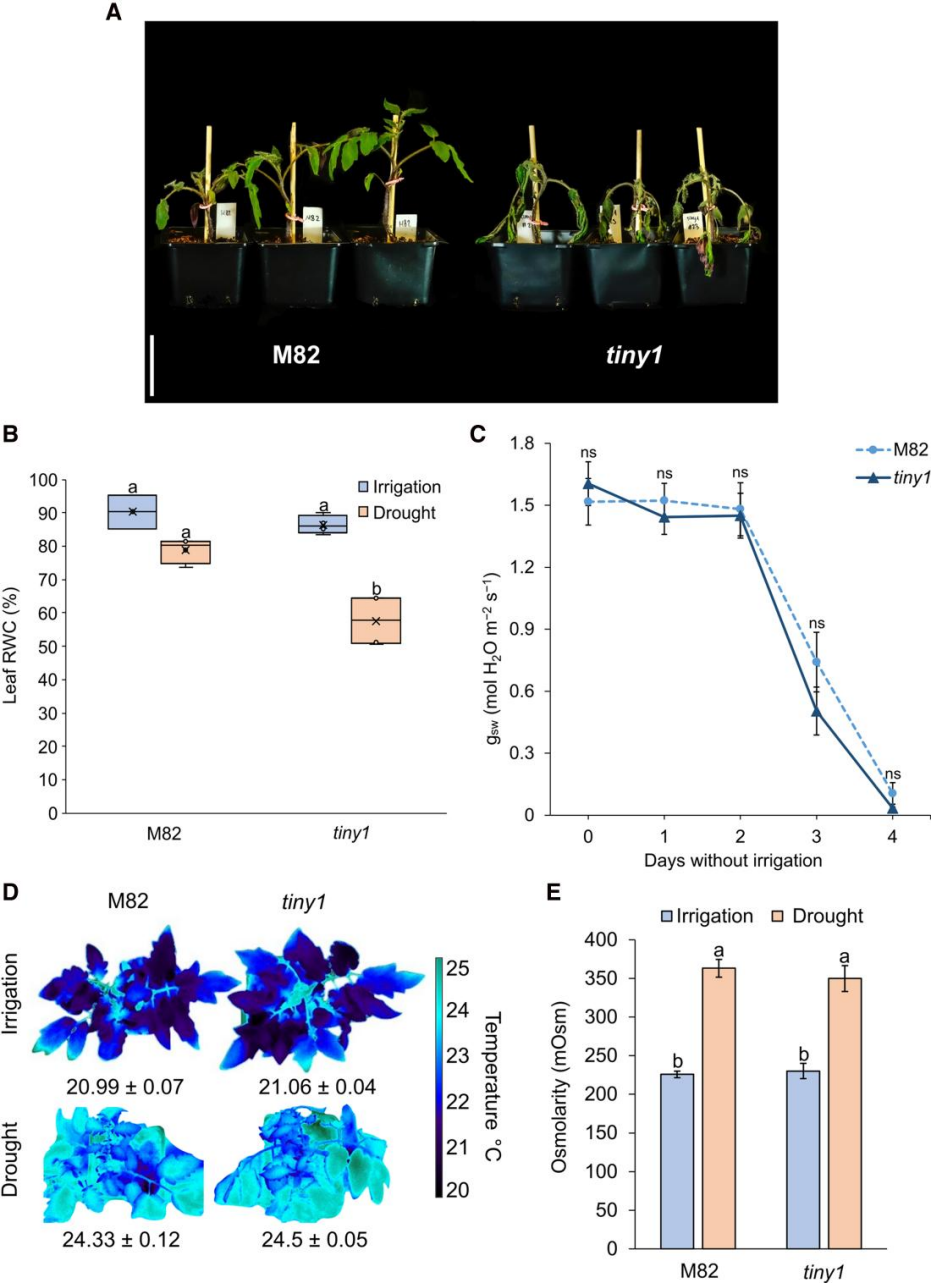
*Tiny1* exhibited faster water loss under drought conditions. (A) Representative M82 and *tiny1-23* plants after 8 d without irrigation. Scale bar=6 cm. (B) Leaf relative water content (RWC, %) of M82 and *tiny1-23* under normal irrigation and 7 d without irrigation. Values are the means ±SE of six biological replicates (terminal leaflets taken from the third leaf below the apex). Different letters above the box plots indicate statistically significant differences (*P*<0.05, Tukey–Kramer HSD). (C) Stomatal conductance (g_sw_) of M82 and *tiny1-23*, measured at 09.00 h over 4 d without irrigation. Data represent the means ±SE of 14 biological replicates for M82 and 19 biological replicates for *tiny1-23*, with three technical replicates per measurement. Statistical significance was determined using Student’s *t*-test (*P*<0.05); ‘ns’ denotes non-significant differences. (D) Thermal imaging of representative M82 and *tiny1-23* plants at 10.00 h under irrigation and 3 d after irrigation was stopped. Numbers below plants are the means ±SE leaf surface temperature taken from all the documented plants, and the values are means of nine biological replicates. (E) Osmolarity of terminal leaflets from the third fully expanded leaf below the apex from M82 and *tiny1-23* plants grown under irrigation or exposed to drought conditions (85% leaf RWC for irrigation and 65% leaf RWC for drought). Data are the means ±SE of five biological replicates per genotype. Different letters above the plots indicate statistically significant differences (*P*<0.05, Tukey–Kramer HSD).

Previously, we demonstrated that tomato *TINY1* is induced by drought. In Arabidopsis, TINY1 regulates stomatal closure and influences the rate of water loss (Xie *et al*., 2019). To assess whether TINY1 activity in tomato also affects water loss, we grew WT M82 and *tiny1* mutant plants under normal irrigation conditions for 5 weeks, after which irrigation was stopped. We then monitored the rate of water loss. *tiny1* plants lost their turgor faster than the M82 (7 d on average into the drought treatment), and the RWC of the leaves at that time was significantly lower in the mutant (Fig. 1A, B).

We then examined stomatal conductance in irrigated plants and during soil dehydration. We did not observe any differences in stomatal conductance between the WT and *tiny1* under either well-watered or drought conditions (Fig. 1C). Transpiration was further assessed using thermal imaging, since evaporative cooling via transpiration is a major driver of leaf temperature regulation. Both genotypes exhibited similar leaf temperatures during soil dehydration, indicating comparable transpiration rates (Fig. 1D). Next, we tested the effect of drought on osmolyte accumulation in the leaves of M82 and *tiny1* plants, under both well-watered conditions and 7 d after irrigation was withheld. No differences were observed between the two genotypes (Fig. 1E). These results do not support the hypothesis that the faster water loss found in *tiny1* is due to changes in stomatal conductance and transpiration rate.

### The increased rate of water loss in *tiny1* is due to larger canopy area

**Fig. 2.**
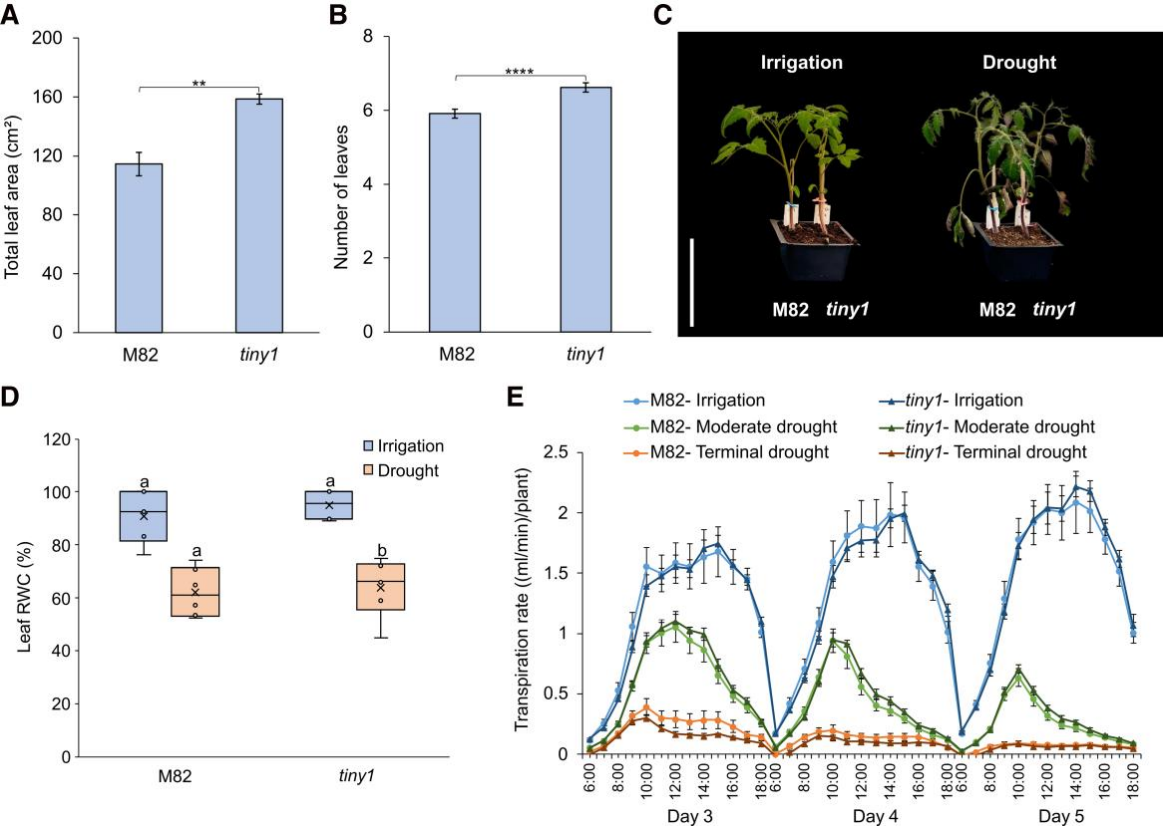
The rapid water loss of *tiny1* under drought is due to its larger leaf area. (A) Total leaf area (cm^2^) of 5-week-old M82 and *tiny1-23* plants. Values are the means ±SE of five plants. (B) Number of leaves in 5-week-old M82 and *tiny1-23* plants. Values are the means ±SE of 40 plants. Asterisks in (A) and (B) represent significant differences between genotypes by Student’s *t*-test (*P*<0.05). (C) M82 and *tiny1-23* plants were grown side by side in the same pot and 6 weeks after irrigation was stopped. Images show representative M82 and *tiny1-23* plants under irrigation and 5 d after irrigation was withheld. Scale bar=15 cm. (D) Leaf RWC (%) of M82 and *tiny1-23* under irrigation and drought conditions (5 d without irrigation). Values are the means ±SE of six biological replicates (terminal leaf taken from the third leaf below the apex). Different letters above the box plots indicate statistically significant differences (*P*<0.05, Tukey–Kramer HSD). (E) Whole-plant transpiration rates of M82 and *tiny1-23* under different irrigation regimes. Transpiration was measured continuously over 72 h (06.00–18.00 h) during days 3, 4, and 5 of the experiment under three irrigation conditions: normal irrigation (control), moderate drought (irrigation of 50% of daily transpiration), and terminal drought (no irrigation). Values represent the means ±SE for seven plants per genotype in the control, nine plants per genotype in the moderate drought, and eight plants per genotype in the terminal drought condition. M82 and *tiny1-*23 plants with the same number of leaves (seven expanded leaves) were placed on lysimeters, and pot weight (pot+soil+plant) was recorded every 3 min.

Larger leaf area leads to increased total transpiration, faster soil dehydration, and earlier loss of turgor. Since it was shown previously that TINY suppresses growth (Xie *et al*., 2019), we compared the leaf area (transpirational surface area) between WT M82 and *tiny1*. M82 and *tiny1* were grown for 6 weeks and then total leaf area and the number of leaves were measured. The foliage area of *tiny1* was larger than that of M82 (Fig. 2A), and the mutant had, on average, one more leaf than M82 (Fig. 2B). To determine if this causes faster water loss and faster wilting of *tiny1*, we grew the mutant and M82 plants side by side in the same pot. This approach eliminates the effect of plant size on the time of turgor loss, since both genotypes are exposed to the same volumetric soil water content throughout the experiment (Shohat *et al*., 2021). After 6 weeks of growth, irrigation was stopped. Six days into the drought treatment, both M82 and *tiny1* plants lost turgor simultaneously and began wilting (Fig. 2C). At this time point, leaf RWC was similar in the two lines (Fig. 2D). We also analyzed whole-plant transpiration in M82 and *tiny1* plants, grown in a greenhouse using an array of lysimeters (Illouz-Eliaz *et al*., 2020). Since there is some degree of variation in plant size (leaf number) in the two genotypes, we were able to select for plants with a similar number of leaves. Plants with seven leaves (M82 and *tiny1*) were grown for 10 d on the lysimeters with irrigation, and then divided into three groups: group 1 continued to receive normal irrigation, group 2 were exposed to moderate drought (received 50% irrigation of their daily transpiration), and for group 3 water supply was terminated. The whole-plant transpiration rate, 3 d after the beginning of the treatments, was similar between M82 and *tiny1* under all tested conditions (Fig. 2E). Together, these results suggest that the accelerated wilting of *tiny1* is likely to be due to its larger leaf area, rather than any inherent differences in dehydration response.

### The effect of TINY1 on global and specific transcriptional activities under drought conditions

**Fig. 3.**
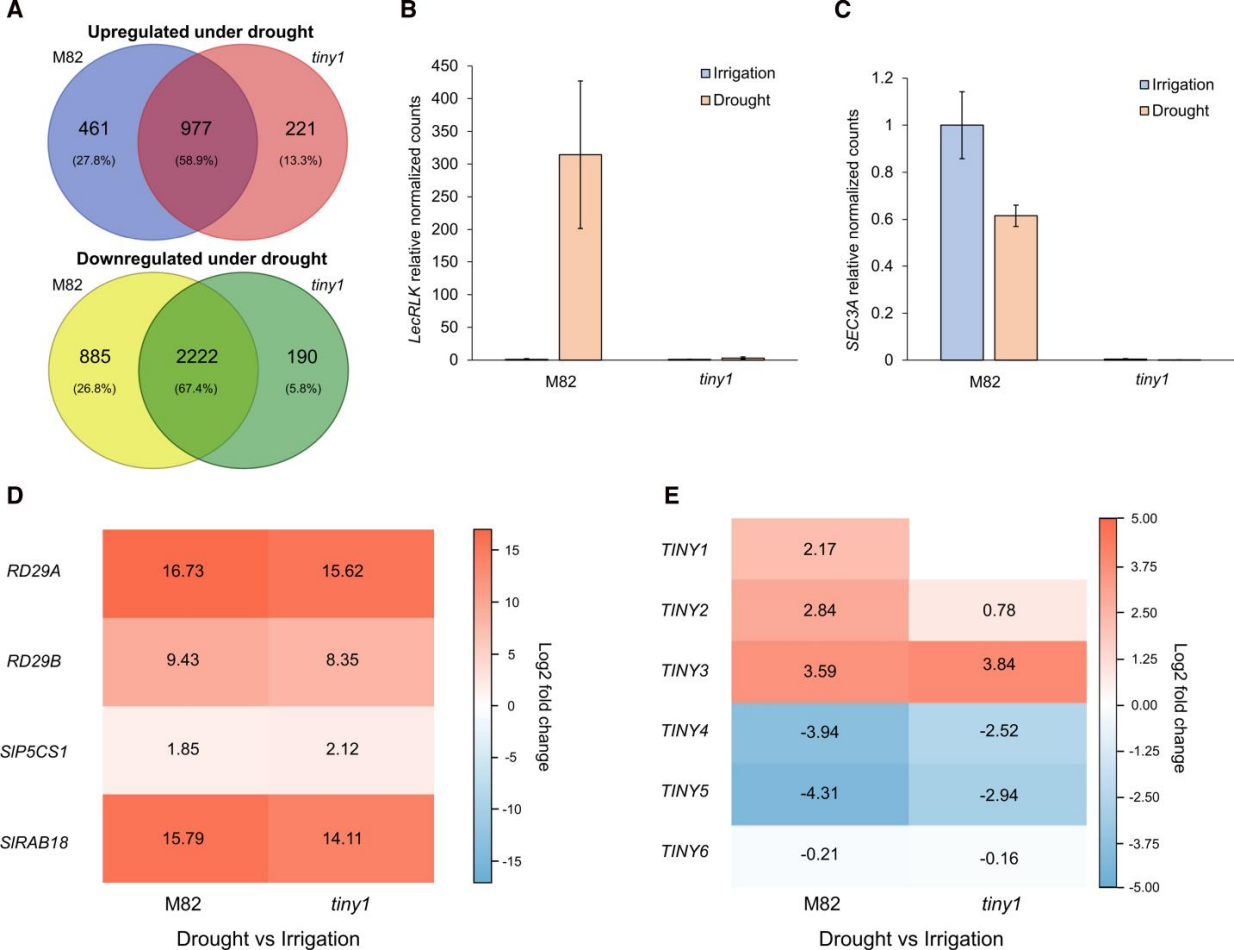
Global and specific transcriptional drought responses in *tiny1* leaves. (A) Venn diagrams showing the overlap of differentially expressed genes (DEGs) between drought (51–55% leaf RWC) and irrigation (80–85% leaf RWC) in M82 and *tiny1-23*. DESeq2 analysis was performed on RNA-seq data to identify up-regulated (top panel) and down-regulated genes (bottom panel). Numbers indicate the number of DEGs, with percentages representing their proportion of the total DEGs. (B) Relative normalized counts of *LECTIN-DOMAIN RECEPTOR-LIKE KINASE* (*LecRLK*) in M82 and *tiny1-23* leaves under irrigation (80–85% leaf RWC) and drought (51–55% leaf RWC). (C) Relative normalized counts of *EXOCYST COMPLEX COMPONENT 3A* (*SEC3A*) in M82 and *tiny1-23* leaves under irrigation (80–85% leaf RWC) and drought (51–55% leaf RWC). (B and C) Values are the means ±SE of three biological replicates. (D) Heatmap of log_2_ fold change (drought versus irrigation) for selected drought-responsive genes in M82 and *tiny1-23*, derived from RNA-seq differential expression analysis. (E) Heatmap of log_2_ fold change (drought versus irrigation) for *TINY* family genes (*TINY1–TINY6*) in M82 and *tiny1-23*, derived from RNA-seq differential expression analysis. (D and E) Drought samples were collected at 51–55% leaf RWC and after irrigation at 80–85% leaf RWC. Colors indicate log_2_ fold change as shown in the color bar on the right (red=up-regulation under drought, blue=down-regulation under drought). Three biological replicates per treatment were used to determine log_2_ fold change.

We next explored the effect of *tiny1* on the leaf transcriptional response under drought by performing RNA-seq analysis. M82 and *tiny1* plants were grown for 5 weeks under normal irrigation conditions, after which irrigation was stopped. Once the plants lost turgor (RWC 50%), terminal leaflets from leaf number 3 (top down) were collected for RNA extraction and RNA-seq analysis. We first examined the global impact of *TINY1* loss of function on drought-responsive gene expression. We applied a 4-fold change threshold (log_2_ fold change ≥2 or ≤ −2) with an adjusted *P*-value (*P*adj) ≤0.05 to control for multiple testing, and excluded genes with low read counts (<30 reads) to ensure detection of robust expression changes. Using these criteria, we identified 4545 differentially expressed genes (DEGs) between irrigated and dehydrated M82 plants, of which 1438 were up-regulated and 3107 were down-regulated. In *tiny1* plants, 3610 DEGs were detected, comprising 1198 up-regulated and 2412 down-regulated genes (Fig. 3A; Supplementary Dataset S1). Most DEGs identified in M82 were also differentially expressed in *tiny1*.

We next searched for putative drought-regulated TINY1 targets. We did not want to exclude genes expressed at a low level or genes that were significantly affected by drought despite having modest fold changes. Thus, we selected genes that were significantly up-regulated in M82 under drought conditions (*P*adj<0.05, without applying a fold change cut-off), but were not affected or were significantly less affected in *tiny1*. This analysis identified 71 candidate genes (Supplementary Dataset S2). The most significantly induced gene under drought in M82, but not in *tiny1*, was *LECTIN-DOMAIN RECEPTOR-LIKE KINASE* (Solyc07g055690) (Fig. 3B), making it a strong candidate for a drought-induced TINY1 target. Among the down-regulated DEGs, we did not identify clear putative TINY1 targets. In addition, independent of drought conditions, comparison of all expressed genes in irrigated M82 plants with those in *tiny1* revealed a strong candidate TINY1 target, *EXOCYST COMPLEX COMPONENT 3A* (*SEC3A*; Solyc07g025170). In the M82 background, *SEC3A* expression was relatively high under irrigated conditions (>700 normalized counts) and was modestly reduced under drought (<30% reduction; Supplementary Fig. S2). In contrast, *SEC3A* expression was undetectable in *tiny1* plants under both irrigated and drought conditions (Fig. 3C; Supplementary Fig. S2).

We also evaluated the effect of drought on known drought-regulated genes *RD29A* (Solyc03g025810), *RD29B* (Solyc01g009660), *SlP5CS1* (Solyc06g019170), and *SlRAB18* (Solyc02g084850) (Nir *et al*., 2017). These genes were similarly affected by drought in M82 and *tiny1* (Fig. 3D). These results suggest that the loss of TINY1 activity has a minor effect on the leaf transcriptional response to drought. We examined the expression of the different *TINY* genes in M82 and *tiny1*. *TINY2* (Solyc03g120840) and *TINY3* (Solyc12g044390) were strongly up-regulated by drought, whereas *TINY4* (Solyc12g008350) and *TINY5* (Solyc08g066660) were down-regulated by drought (Fig. 3E). The expression of *TINY6* (Solyc01g090560) was not affected. In *tiny1*, *TINY2*, *TINY4*, and *TINY5* showed a reduced response to drought compared with M82, whereas *TINY3* and *TINY6* exhibited responses similar to M82. Although loss of *TINY1* activity did not enhance the drought-induced up-regulation of *TINY2* and *TINY3*, the lack of an observable drought-response phenotype—such as changes in stomatal activity and gene expression—in the *tiny1* mutant may result from functional redundancy with these genes.

### TINY1 suppressed embryonic and early seedling development

**Fig. 4.**
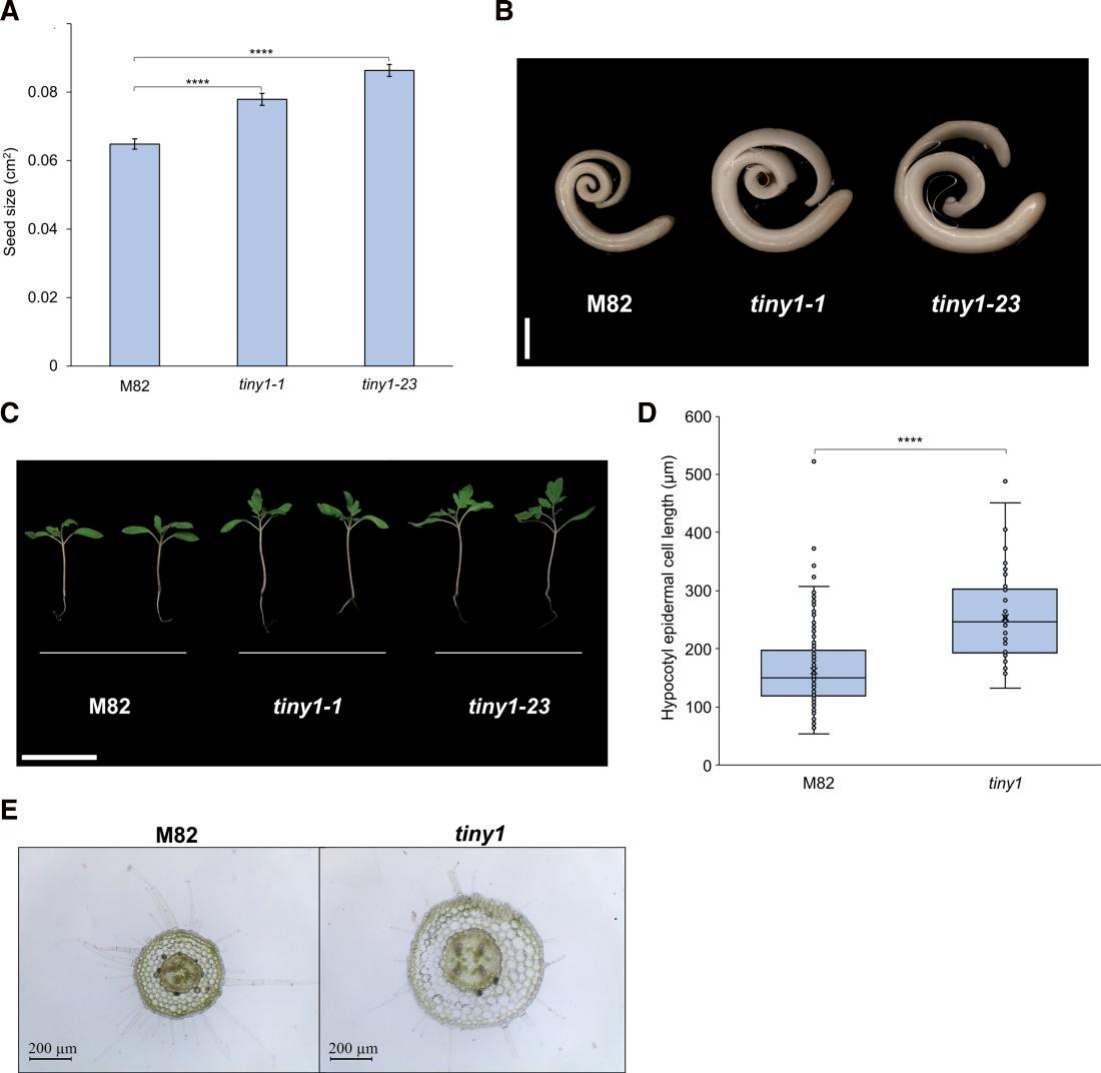
TINY1 suppressed embryo and seedling growth. (A) M82, *tiny1-1*, and *tiny1-23* seed size (cm^2^). Values are the means ±SE of 30 seeds. Asterisks indicate significant differences between genotypes by Dunnett test (*P*<0.05). (B) Embryos of M82, *tiny1-1*, and *tiny1-23* extracted from dry seeds. Scale bar=1 mm. (C) Representative seedlings of M82, *tiny1-1*, and tiny*1-23* 18 d after germination. Scale bar=5 cm. (D) Hypocotyl epidermal cell length measured in 2-week-old seedlings of M82 and *tiny1-23*. Values are the means ±SE of 230 cells of M82 and 300 cells of *tiny1*. Asterisks represent significant differences between genotypes by Student’s *t*-test (*P*<0.05). (E) Representative cross-sections of hypocotyls from 20-day-old M82 and *tiny1-23* seedlings. Scale bar=200 µm.

To determine at which developmental stage the loss of TINY1 activity affects growth, we followed the rate of leaf production and found that *tiny1* developed the first true leaf earlier than M82, but later the rate of leaf production was similar (Supplementary Fig. S3). These results suggest that the effect of TINY1 on growth is restricted to early stages of plant development. We found that the seeds of *tiny1* (lines *tiny1-1* and *tiny1-23*) were larger than those of M82 (Fig. 4A; Supplementary Fig. S4). We therefore extracted the embryos from the seeds and measured their size. The embryos of *tiny1* were much larger than those of M82 (Fig. 4B; Supplementary Fig. S5). We also examined the seedlings and found that 2 weeks after germination the hypocotyl of *tiny1* was longer than in M82 (Fig. 4C; Supplementary Fig. S6). Microscopic analyses of the epidermal cells and cross-sections of the hypocotyl suggest the TINY1 affects cell elongation and expansion rather than cell division (Fig. 4D, E; Supplementary Fig. S7A, B). Together, these results suggest that TINY1 acts as a growth suppressor during the early stages of plant development.

TINY1 expression is induced by drought. Since we found evidence that TINY1 acts as an embryonic growth suppressor, we hypothesized that its role in drought conditions is to suppress plant growth. We therefore grew M82 and *tiny1* plants under mild drought conditions that reduce growth rates. The plants were grown for 3 weeks under regular irrigation, after which irrigation was stopped. When the plants began to lose turgor, 40% of the normal irrigation volume was added. This treatment was repeated for five cycles, lasting ∼35 d. At the beginning of the drought treatment and just before each subsequent irrigation, stem length was measured and leaf number counted. At the end of the experiment, leaf area was measured. Drought treatment inhibited stem elongation and leaf production, but the effect was similar in M82 and *tiny1* (Supplementary Fig. S8A, B). The total leaf area was strongly reduced by drought, but again the loss of TINY1 activity did not affect this growth rate (Supplementary Fig. S8C). This suggests that either TINY1 has no role in regulating plant growth under water-limited conditions or its function is completely redundant with the activity of other TINY proteins.

### TINY1 attenuated gibberellic acid activity in the maturing embryo

**Fig. 5.**
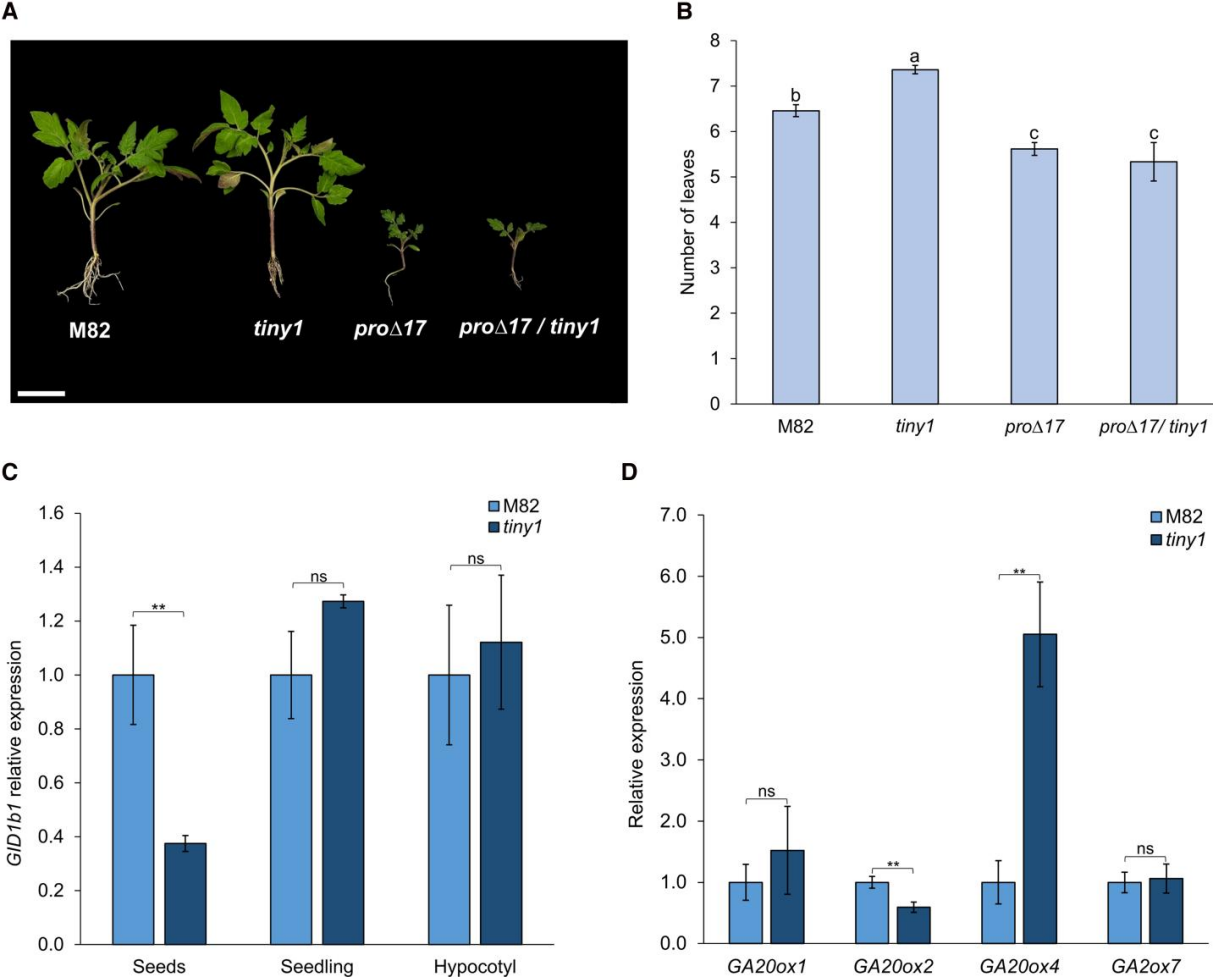
TINY1 repressed embryo growth via the GA pathway. (A) Representative seedlings of M82, *tiny1-23*, 35S:*proΔ17*, and 35S:*proΔ17*/*tiny1-*23. (B) Number of leaves in 30-day-old M82, *tiny1-23*, 35S:*proΔ17*, and 35S:*proΔ17/tiny1-23* plants. Data are the means ±SE of 37 (M82), 33 (*tiny1-23*), 13 (35S:*proΔ17*), and 6 (35S:*proΔ17*/*tiny1-23*) plants. Different letters indicate statistically significant differences between lines (*P*<0.05, Tukey–Kramer HSD test). (C) Relative expression (RT–qPCR) of *GID1b1* in seeds harvested from red-ripe fruit, in 1-week-old seedlings and in hypocotyls of M82 and *tiny1-23*. Expression levels are normalized to M82 seeds and presented as the means ±SE of a minimum of four replicates. (D) Relative expression (RT–qPCR) of GA biosynthesis and deactivation genes (*GA20ox1*, *GA20ox2*, *GA20ox4*, and *GA20ox7*) in seeds harvested from red-ripe fruit of M82 and *tiny1-23*. Expression levels are normalized to M82 and values are the means ±SE of four biological replicates. Asterisks in (C) and D) indicate significant differences between genotypes (*P*<0.05, Student’s *t*-test); ‘ns’ denotes no significant difference.

Previous studies in tomato suggest that TINY1 regulates GA biosynthesis in leaves via the inhibition of *GA20ox1*, *GA20ox2*, and *GA20ox4* expression (Li *et al*., 2012), and GA catabolism in guard cells by the induction of *GA2ox7* (Shohat *et al*., 2021). We therefore generated *tiny1* homozygous plants on the *PROCERA* (*pro*)*Δ17* background (Nir *et al*., 2017). *proΔ17* plants overexpress a gain-of-function stable version of the DELLA protein PRO that constitutively inhibits GA activity. The double mutant *tiny1 proΔ17* seedlings exhibited the dwarf phenotype (short hypocotyl) of *proΔ17*, and leaf number was the same as in M82 and *proΔ17* but less than in *tiny1* (Fig. 5A, B; Supplementary Fig. S9), suggesting that PRO is epistatic to TINY1 and supporting the hypothesis that TINY1 regulates growth via its effect on GA biosynthesis/catabolism. To test if GA activity increases in the *tiny1* mutant, we analyzed the expression of *GID1b1* (GA receptor gene). *GID1b* expression is inhibited by increased GA activity due to a feedback response (Illouz-Eliaz *et al*., 2019). The expression of *GID1b1* was analyzed in seeds extracted from red fruits, in seedlings following germination, and in elongating hypocotyls 2 weeks after germination. *GID1b1* expression was inhibited in *tiny1* (compared with M82) seeds but not in seedlings or in elongating hypocotyl (Fig. 5C), suggesting increased GA activity in the mutant embryos, but not later during seedling development. We then analyzed the candidate targets of TINY1: *GA20ox1*, *GA20ox2*, *GA20ox4*, and *GA2ox7* in seeds extracted from red fruits. The expression of *GA20ox4*, but not of the other examined genes, was significantly affected (up-regulated) by *tiny1* (Fig. 5D–G). To verify that these results are reliable, we used an additional reference gene (*TIP41*) acting in tomato seeds (Shohat *et al*., 2023), and found similar results (Supplementary Fig. S10). We further analyzed other *GA20ox*, *GA3ox*, and *GA2ox* genes but none of them was affected significantly by the mutation (Supplementary Fig. S11). These results suggest that TINY1 suppresses the expression of *GA20ox4* in the embryo to reduce GA production.

## Discussion

DREB transcription factors play an important role in plant responses to various abiotic and biotic stresses, including drought. Many studies have shown their effect on drought avoidance responses (stomatal closure and growth suppression under drought) and drought tolerance (regulation of drought-responsive genes) (Lata and Prasad, 2011; Sarkar *et al*., 2019). This family of proteins is divided into six subfamilies (A1–A6). A subgroup of subfamily A4 is named TINY (Nakano *et al*., 2006). TINY proteins seem to regulate both growth and abiotic responses. TINY1-overexpressing Arabidopsis and tomato plants are semi-dwarf (Wilson *et al*., 1996; Li *et al*., 2012). Moreover, the triple Arabidopsis mutant *tiny1tiny2tiny3* has larger leaves and longer petioles (Xie *et al*., 2019). In both plants, overexpression of TINY1 improved drought tolerance due to increased drought-responsive gene expression and hypersensitivity to ABA-mediated stomatal closure (Sun *et al*., 2008; Xie *et al*., 2019).

We found that the loss of the *TINY1* gene in tomato resulted in early wilting when exposed to water-limited conditions (Fig. 1A, B). However, this rapid water loss was not associated with increased stomatal conductance or impaired osmotic adjustment (Fig. 1C–E); but rather with an increased leaf area which led to greater total transpirational surface (Fig. 2A, B). The *tiny1* mutant produced its first true leaf earlier than the control M82, resulting in the development of, on average, one additional leaf during early vegetative growth before the onset of its first inflorescence (Fig. 2). RNA-seq analysis of leaves taken from well-watered and drought-treated M82 and *tiny1* plants revealed similar expression of known drought-responsive genes (Fig. 3D). This, however, does not necessarily suggest that TINY proteins have no effect on drought-related gene expression. Three *TINY* genes were up-regulated by drought in leaves, *TINY1*, *TINY2*, and *TINY3* (Fig. 3E); it is therefore possible that *TINY2/3* compensated for the loss of *TINY1* in the regulation of drought-induced molecular responses in leaves, due to functional redundancy. We identified, however, a potential drought-induced TINY1 target: *LECTIN-DOMAIN RECEPTOR-LIKE KINASE*, whose expression was strongly up-regulated by drought in M82 leaves but not in *tiny1* (Fig. 3B). Members of this receptor family have been shown to play key roles in plant development as well as in responses to both abiotic and biotic stresses (Vaid *et al*., 2013; Sun *et al*., 2020). Additionally, our analysis revealed the tomato exocyst subunit *SEC3A* as another potential TINY1 target in leaves (Fig. 3C). Although *SEC3A* expression was not affected by drought, it was completely suppressed in all *tiny1* samples, regardless of water availability. Previous studies have implicated SEC3A in several key processes, including cell growth (Hála *et al*., 2008), embryo development (Zhang *et al*., 2013), and pathogen defense (Du *et al*., 2018). Based on these findings, the loss of exocyst activity would be expected to result in smaller embryo and shorter hypocotyls. However, *tiny1* mutants exhibited larger embryos and longer hypocotyls, thus the developmental role of TINY1 in regulating *SEC3A* remains unclear.

The only clear and consistent phenotypes we observed in *tiny1* were larger seeds and embryos, longer hypocotyls, and early emergence of the first true leaf (Fig. 4A–D). At later stages of seedling and plant development, we did not find differences in plant growth rate and development. As mentioned above, the effect of TINY proteins on plant growth was shown before (Wilson *et al*., 1996; Li *et al*., 2012; Xie *et al*., 2019). Here we show that in tomato its effect on plant size resulted from its regulation of embryo and early seedling development. Xie *et al*. (2019) proposed that the Arabidopsis TINY proteins are regulated by BR and, in turn, modulate BR signaling. Under drought conditions, the kinase BIN2 phosphorylates and stabilizes TINY proteins. The accumulated TINY then interacts with and inhibits the BR-responsive transcription factor BES1, thereby repressing BR-mediated growth and activating drought-responsive genes. In contrast, under normal conditions, active BR signaling promotes the degradation of TINY proteins, which promotes growth and suppresses stress-related gene expression. In tomato, on the other hand, TINY1 overexpression suppressed GA biosynthesis (Li *et al*., 2012), suggesting that the effect of TINY on growth is via the GA response pathway. Our results also suggest that TINY1 promotes embryo growth through the GA pathway. Loss of TINY1 function strongly induced the expression of the GA biosynthesis gene *GA20ox4* in maturing seeds (Fig. 5D). Elevated GA activity typically triggers a feedback response that, in tomato, includes suppression of the GA receptor gene *GID1b1* (Illouz-Eliaz *et al*., 2019). The reduced expression of *GID1b1* in *tiny1* maturing seeds further supports the notion of increased GA activity (Fig. 5C). In addition, the excess hypocotyl elongation and the early leaf growth phenotype observed in *tiny1* were suppressed in the presence of a stabilized DELLA protein (Fig. 5A, B), suggesting that TINY1 acts upstream of DELLA in the GA pathway. Although DELLA proteins also influence the BR pathway (Shani *et al*., 2024), our collective findings indicate that in developing embryos, TINY1 primarily inhibits the GA pathway to suppress growth. While *tiny1* mutants displayed enhanced embryo size, longer hypocotyls, and accelerated emergence of the first true leaves, we detected altered GA activity only in the maturing embryo. This may suggest that GA induces stable epigenomic changes in growth-related genes that persist following seed dormancy and germination. Alternatively, TINY1 may act via different pathways to regulate embryo growth and hypocotyl elongation; for example, in the embryo, it suppresses GA activity and, in the hypocotyl, BR activity.

To conclude, tomato TINY1 probably has two different functions; as an embryonic growth suppressor and an as yet unknown function in leaves under drought. The inhibition of embryo growth and hypocotyl elongation by TINY1 may contribute to improved seedling resilience following germination under changing environments.

## Supplementary Material

erag054_Supplementary_Data

## Data Availability

All data can be found in the manuscript and in the online Supplementary data. Sequence data (RNA-seq) from this article were deposited in NCBI’s Sequence Read Archive (SRA) under the BioProject accession number PRJNA1283911 and SRA accession number SRO595868 (https://www.ncbi.nlm.nih.gov/bioproject/PRJNA1283911).

## Supplementary data

The following supplementary data are available at *JXB* online.

Fig. S1. Sequence analysis of the *tiny1-23* allele.

Fig. S2. Validation of *EXOCYST COMPLEX COMPONENT 3A* (*SEC3A*) expression in M82 and *tiny1.*

Fig. S3. Rate of leaf production in M82 and *tiny1-23* plants.

Fig. S4. Seeds of M82 and *tiny1* mutants.

Fig. S5. M82 and *tiny1* embryo size.

Fig. S6. M82 and *tiny1* hypocotyl length.

Fig. S7. The loss of TINY1 activity promoted hypocotyl cell elongation and expansion.

Fig. S8. The loss of TINY1 activity did not promote plant growth under drought.

Fig. S9. Stable DELLA protein inhibited the effect of *tiny1* on seedling growth.

Fig. S10. Normalization using the reference gene *TIP41* yielded similar results to those obtained with *ACTIN* in RT–qPCR analyses of gene expression in M82 and *tiny1* seeds.

Fig. S11. Expression of GA biosynthesis and catabolism genes in seeds of M82 and *tiny1*.

Table S1. Primers used in this study.

Dataset S1. Differentially expressed genes (DEGs) between irrigation and drought conditions in M82 and *tiny1*.

Dataset S2. Differentially expressed drought-induced genes between M82 and *tiny1*.

## Abbreviations

DEG: differentially expressed gene
DREB: dehydration-responsive element-binding
RWC: relative water content
