# Supplementary material for: Tomato drought-responsive transcription factor TINY1 suppresses embryonic growth
Source: J Exp Bot. 2026 Feb 6;77(12):3700–11. doi: 10.1093/jxb/erag054 (PMC13293088; doi:10.1093/jxb/erag054)
Supplement: erag054_Supplementary_Data [file erag054_supplementary_data.zip › jexbot316968-file001.pdf]

Supplementary Data

Supplementary Figures

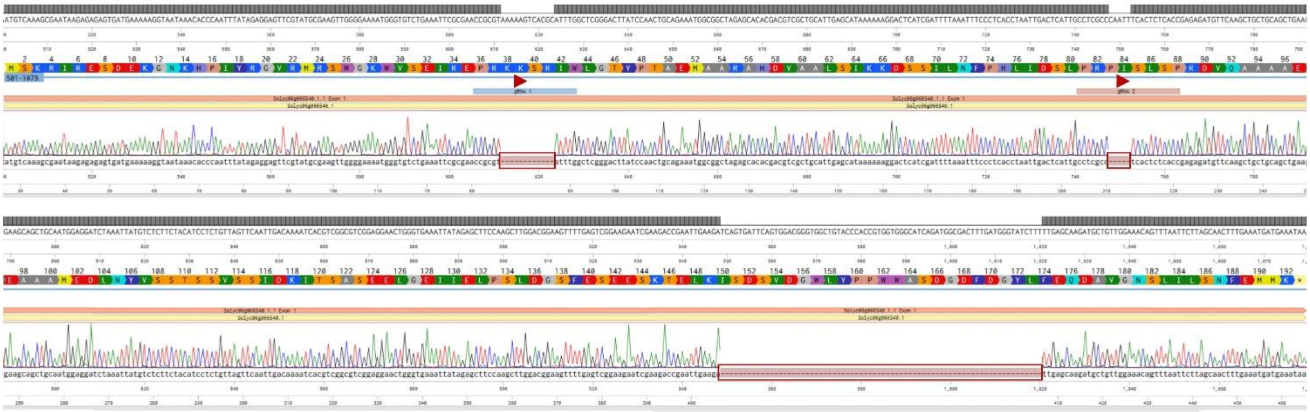

Supplementary Figure S1. Sequence analysis of the *tiny1-23* allele. Wild-type (upper) and CRISPR-derived *tiny1-23* allele (lower), showing 12,5 and 72 bp deletions. Red arrows indicate the position of the RNA guides in the coding sequence of *TINY1*. Also shown the chromatograms and the nucleotide sequences including the deletion sites (highlighted red).

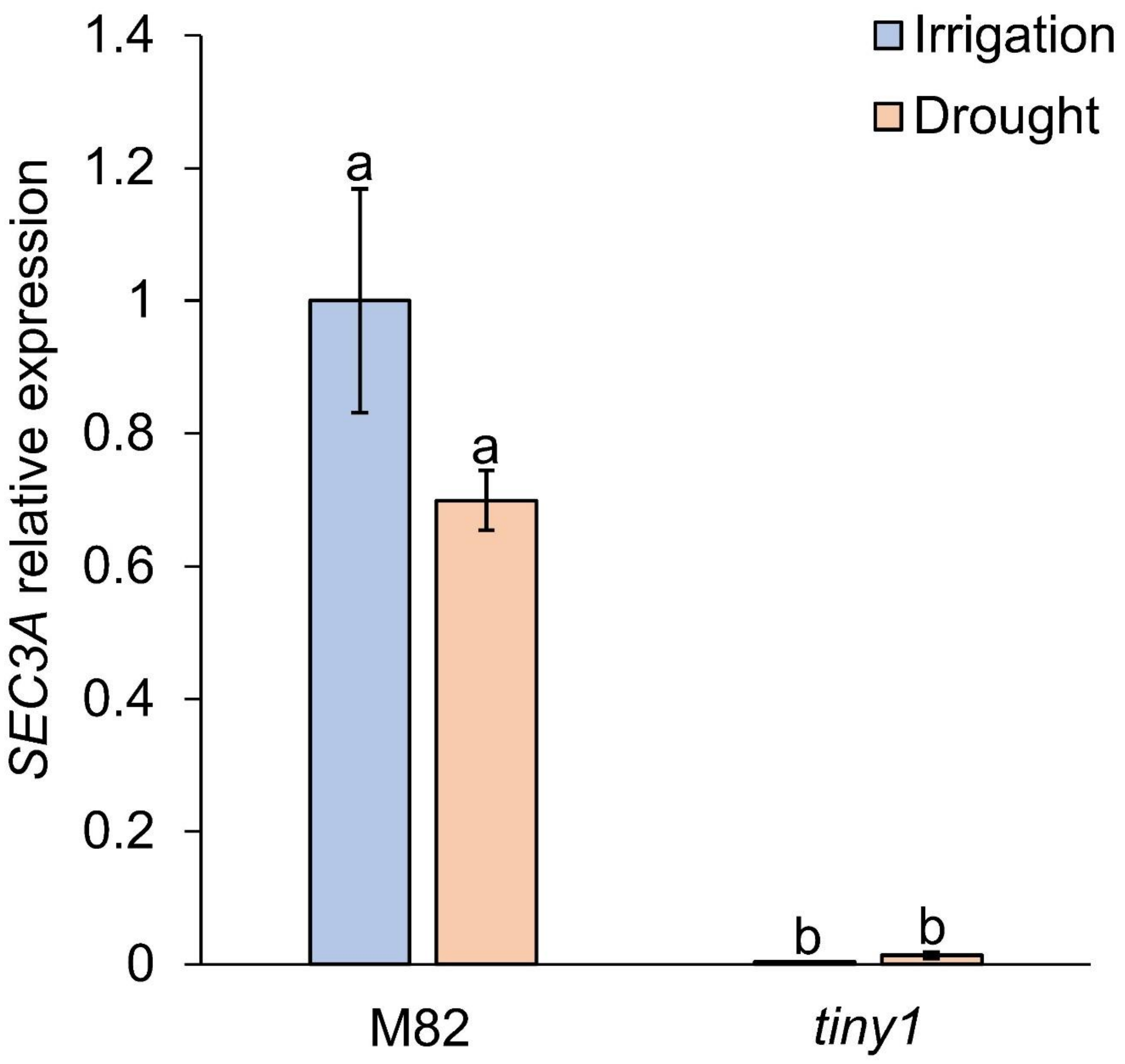

Supplementary Figure S2. Validation of *EXOCYT COMPLEX COMPONENT 3A* (*SEC3A*) expression in M82 and *tiny1*. RT-qPCR analysis of SEC3A in M82 and *tiny1-23* leaves under irrigation (85% leaf RWC) and drought (52% leaf RWC). Values are means± SE of four biological replicates. Different letters indicate statistically significant differences ($P < 0.05$, Tukey-Kramer HSD).

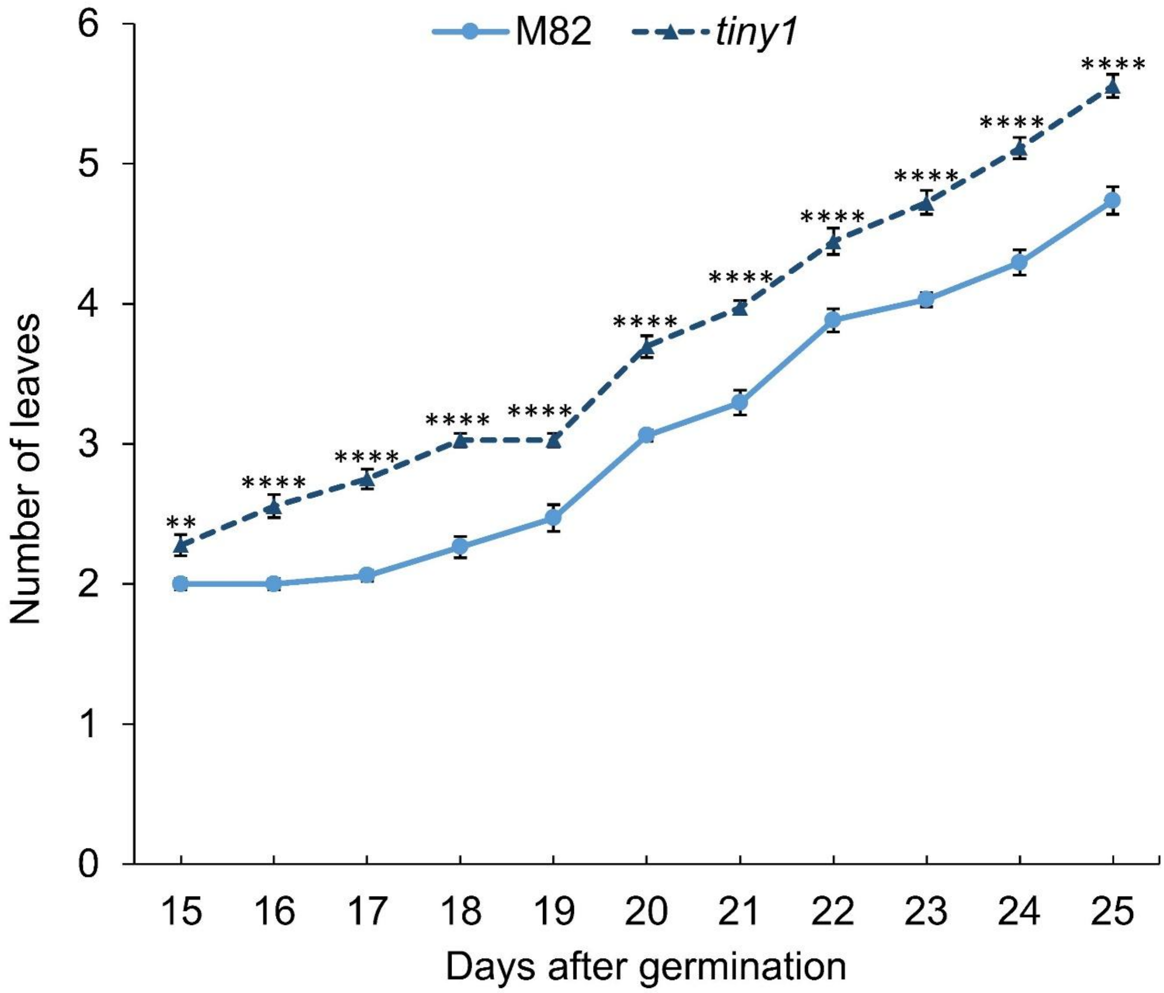

Supplementary Figure S3. Rate of leaf production in M82 and *tiny1* plants. Leaves were counted daily in irrigated M82 and *tiny1*-23 plants. Data are means± SE of 36 plants. Asterisks indicate significant differences ($P < 0.05$, Student's *t*-test).

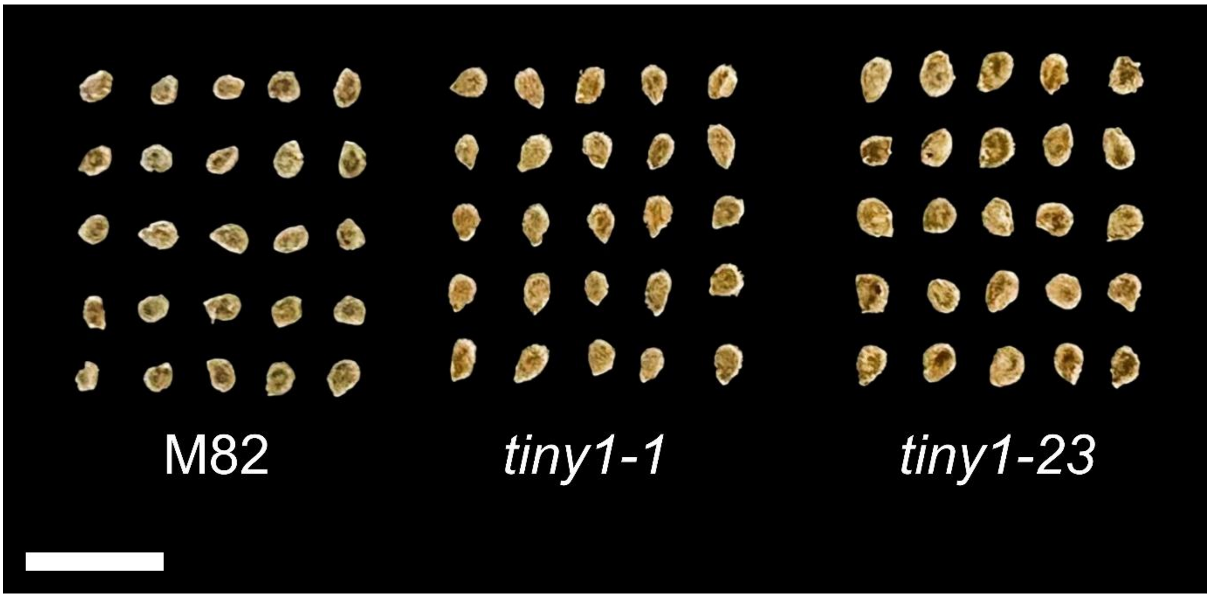

Supplementary Figure S4. Seeds of M82 and *tiny1* mutants. M82, *tiny1-1* and *tiny1-23* representative seeds. Scale bar= 1 cm.

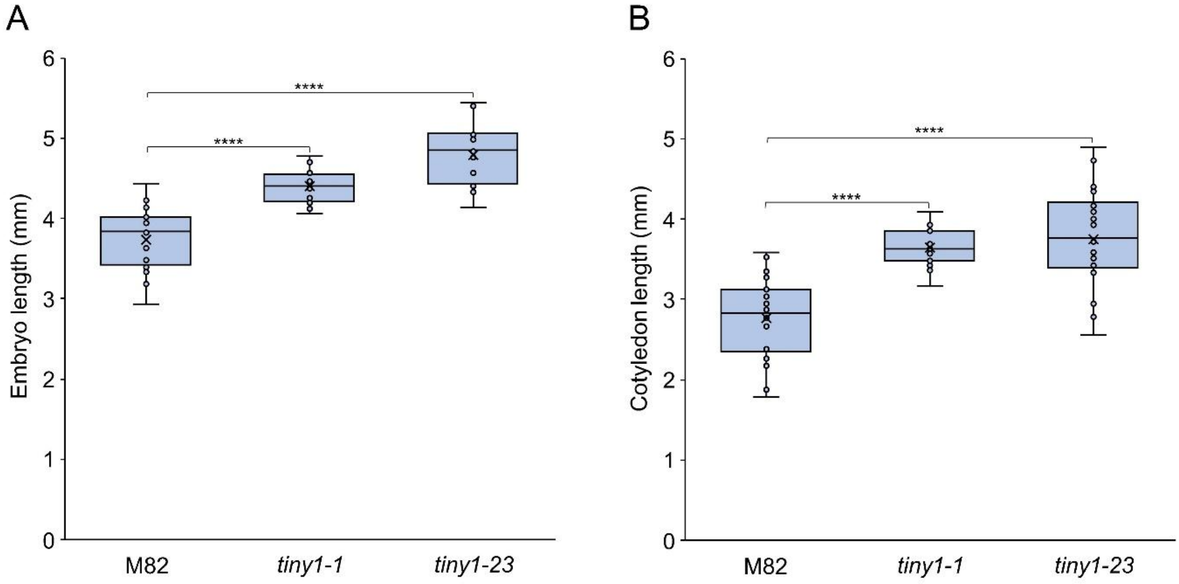

Supplementary Figure S5. M82 and *tiny1* embryo size. Embryos were extracted from dry M82, *tiny1-1* and tiny*1-23* seeds. **A**. Length from root tip to shoot apical meristem. **B**. Length of cotyledons. Values are means± SE of 20 embryos. Statistical significance was determined using the Dunnett test ($P < 0.05$).

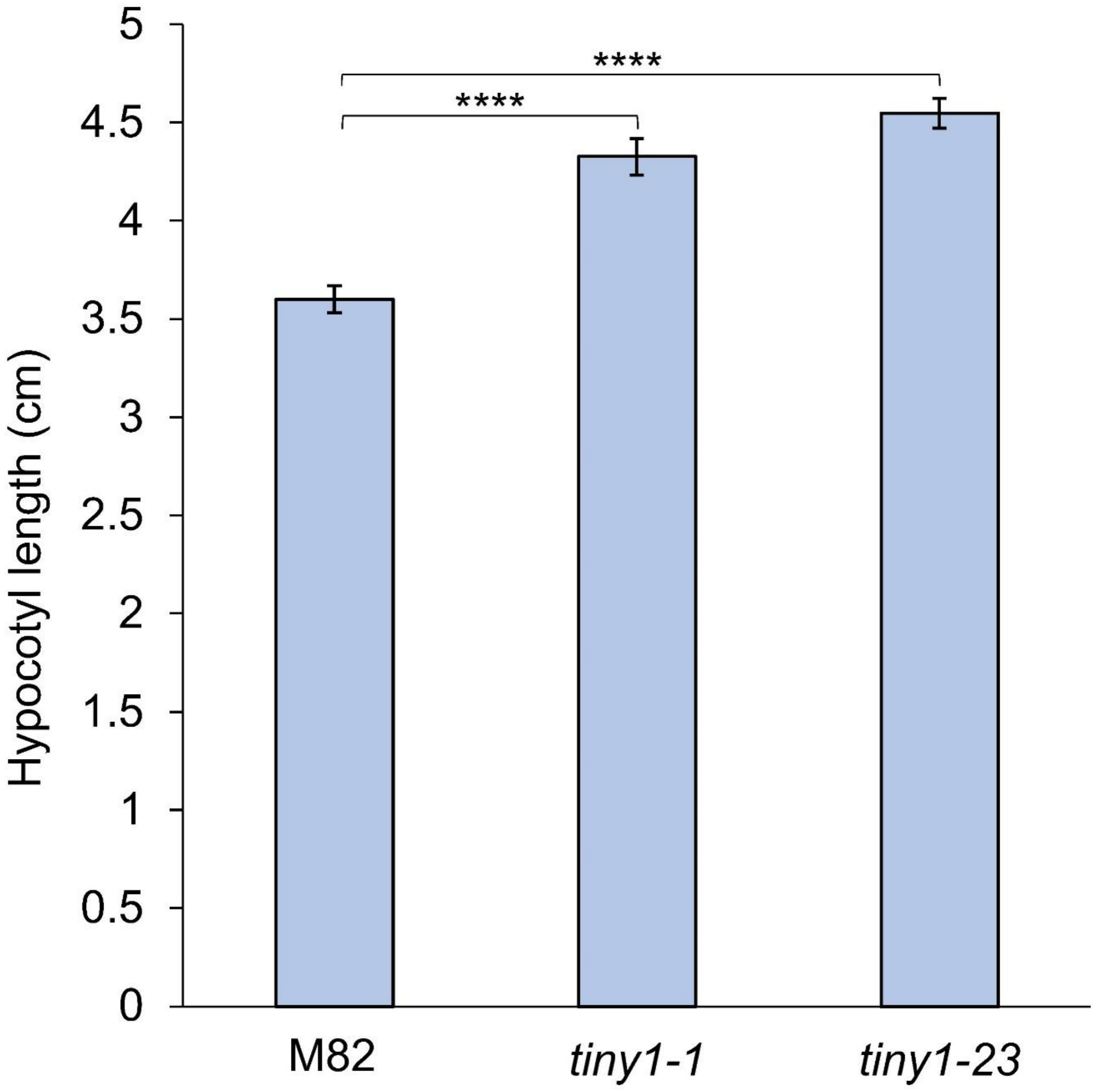

Supplementary Figure S6. M82 and *tiny1* hypocotyl length. Hypocotyl length of M82, *tiny1-1* and *tiny1-23*. Values are means± SE of 30 biological replicates for M82, 23 for tiny-1 and 27 for tiny1-23. Significant differences between respective genotypes were determined using Dunnett test ($P < 0.05$).

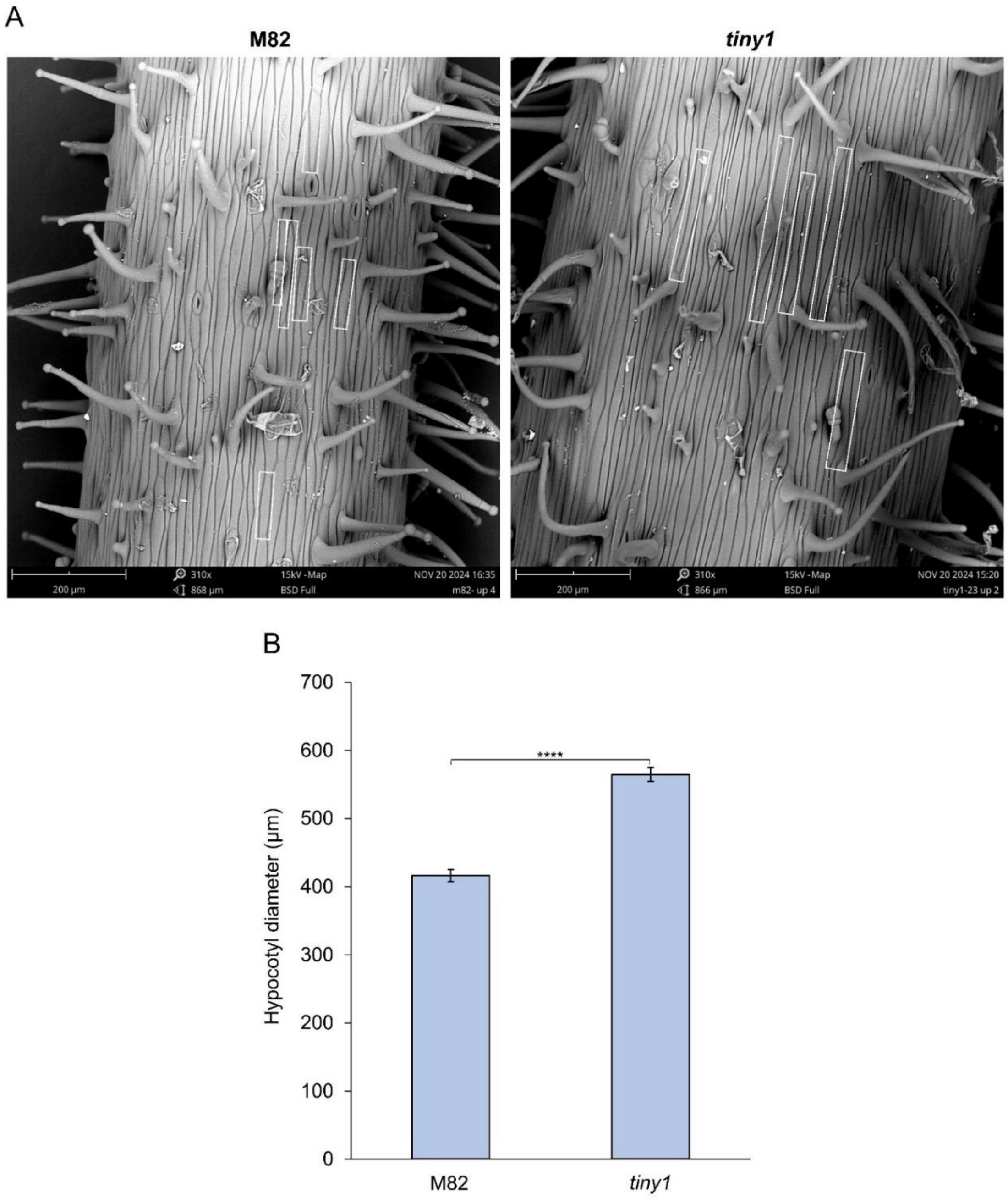

Supplementary Figure S7. The loss of TINY1 activity promoted hypocotyl cell elongation and expansion. **A**. Representative images taken from scanning electron microscopy (SEM) analyses of hypocotyl epidermal cells of 2-week-old M82 and *tiny1-23* seedlings. Cells are marked with a white rectangle. Scale bar= 200 µm. **B**. Hypocotyl diameter of M82 and *tiny1-23* 20-day-old seedlings analyzed by light microscopy of cross-sections. Values are means± SE of 15 biological replicates.

Asterisks represent significant differences between genotypes determined by Student's t-test (P < 0.05).

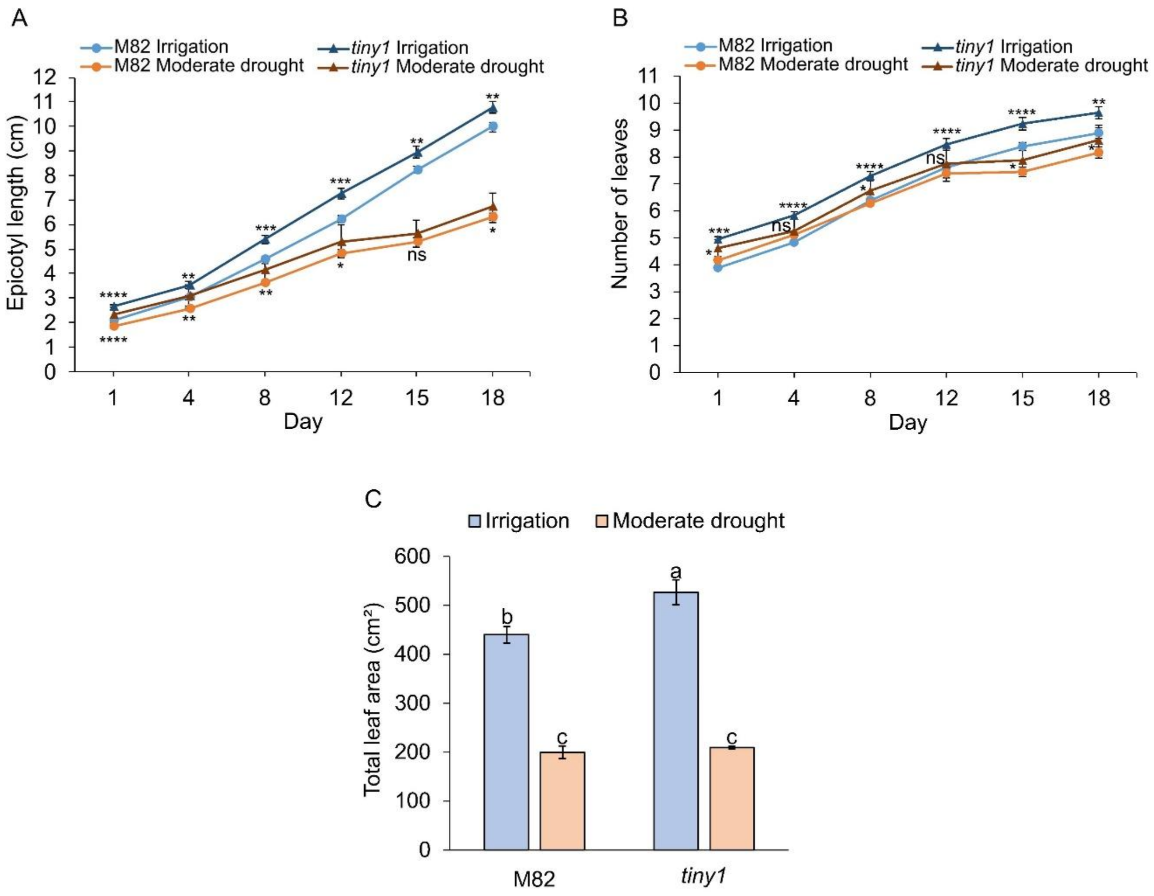

Supplementary Figure S8. The loss of TINY1 activity did not promote plant growth under drought. **A**. Rate of epicotyl elongation in irrigated and moderate drought treated M82 and tiny1-23 plants. Values are means± SE of 18 biological replicates. **B**. Number of leaves as in A. Asterisks represent significant differences between genotypes in each determined by Student's t-test (P < 0.05). ns- non-significant differences. **C**. Total leaf area (cm²) in 45-day-old M82 and *tiny1-23* plants grown under normal irrigation or moderate drought. Values are means± SE of six biological replicates Different letters indicate statistically significant differences (P < 0.05, Tukey-Kramer HSD).

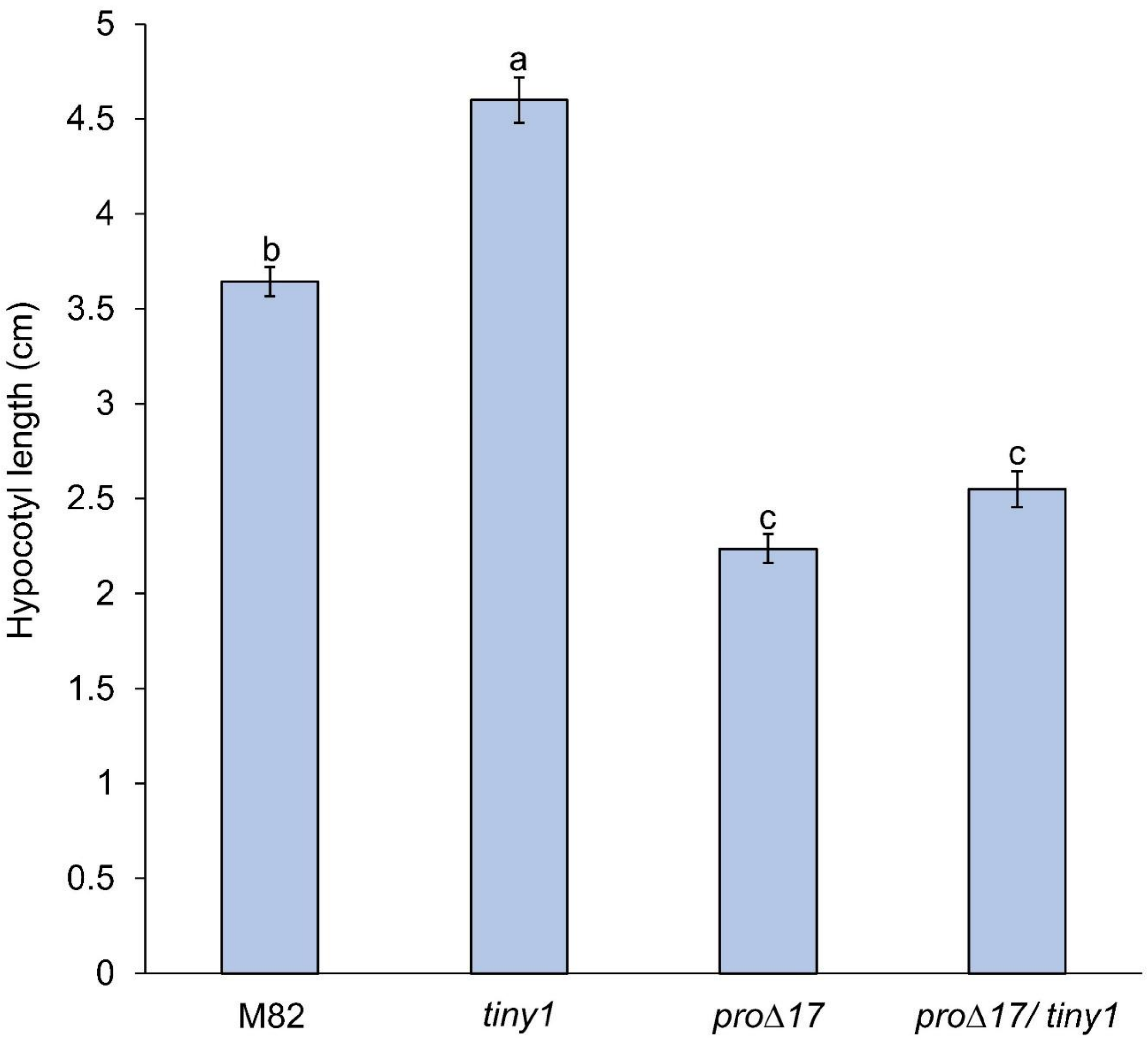

Supplementary Figure S9. Stable DELLA protein inhibited the effect of tiny1 on seedling growth. Hypocotyl length in 35-day-old M82, *tiny1-23*, *35S:proΔ17*, and *35S:proΔ17/tiny1-23* plants. Values are means ± SE of 38, 33, 8 and 6 biological replicates of 82, *tiny1-23*, *35S:proΔ17* and *35S:proΔ17/tiny1-23*, respectively. Different letters indicate statistically significant differences ($P < 0.05$, Tukey-Kramer HSD).

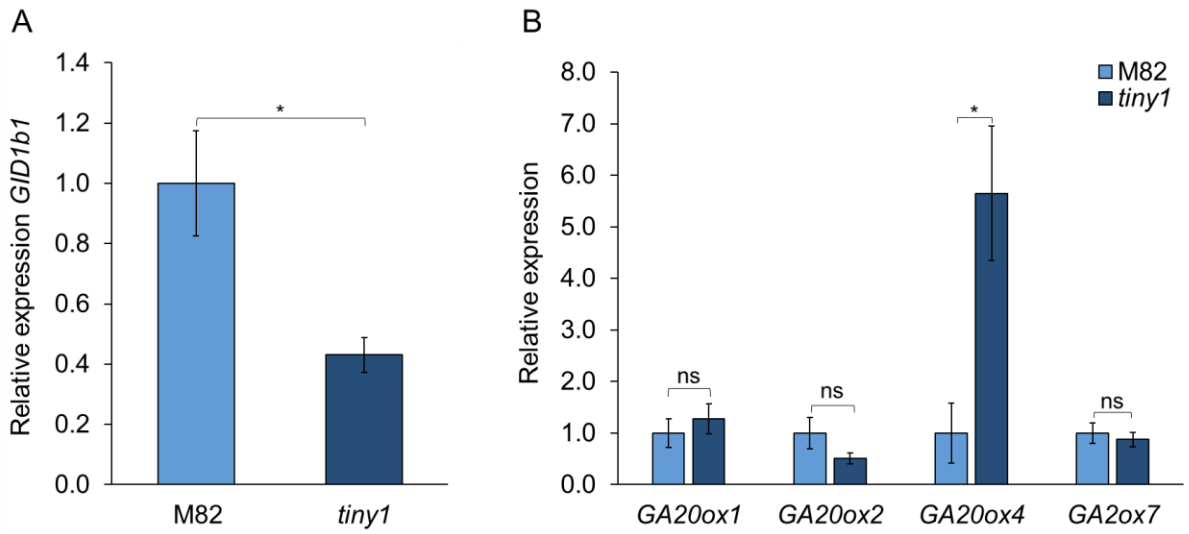

Supplementary Figure S10. Normalization using the reference gene *TIP41* yielded similar results to those obtained with *ACTIN* in RT-qPCR analyses of gene expression in M82 and *tiny1* seeds. **A**. Relative expression of *GID1b1* in seeds harvested from red-ripe fruit of M82 and *tiny1-23*. **B**. Relative expression of GA biosynthesis and deactivation genes (*GA20ox1*, *GA20ox2*, *GA20ox4* and *GA20ox7*) in seeds harvested from red-ripe fruit of M82 and *tiny1-23*. Relative expression was calculated by dividing the expression level of *GID1b1 or GA20ox1/2/4 or GA2ox7* to *TIP41*. Expression levels are normalized to M82 seeds and presented as means ± SE of 4 replicates.

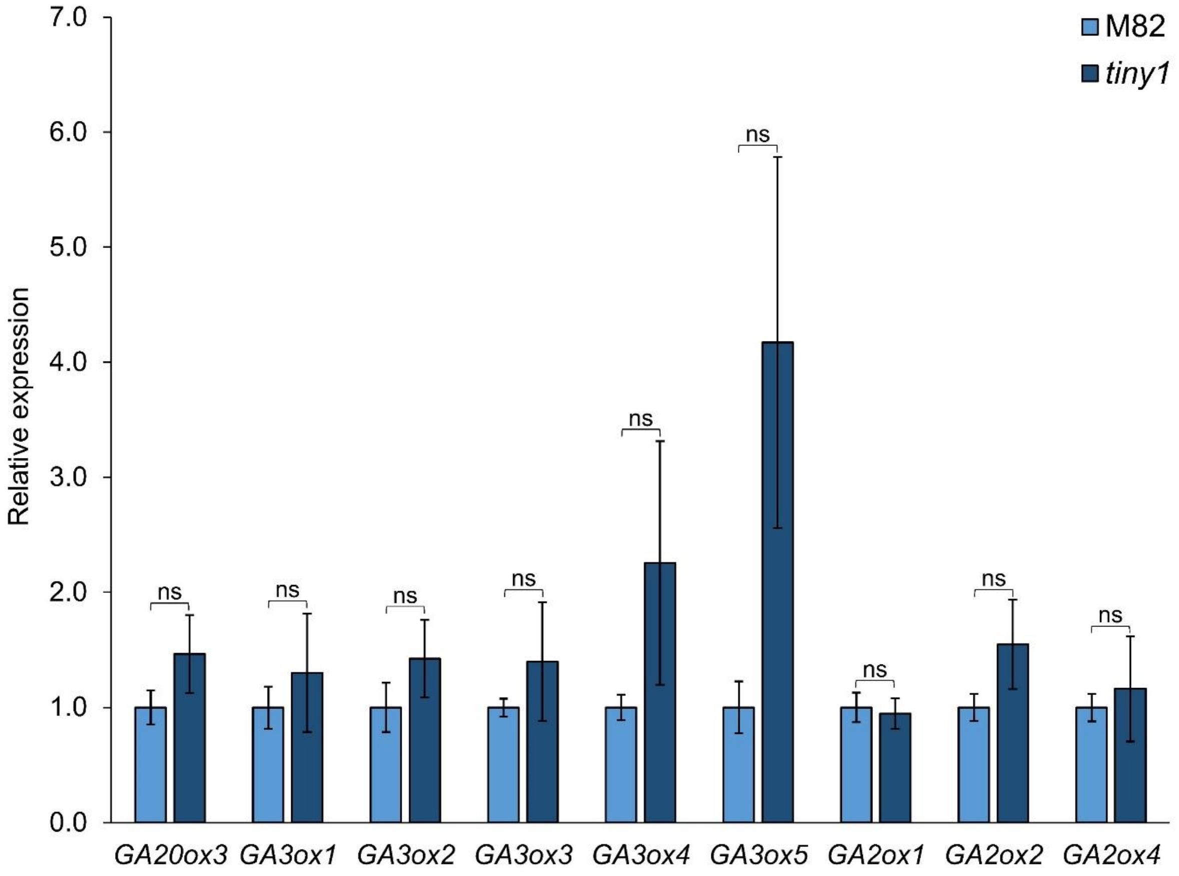

Supplementary Figure S11. Expression of GA biosynthesis and catabolism genes in seeds of M82 and *tiny1*. RT-qPCR analysis of GA biosynthesis (*GA20ox* and *GA3ox*) and deactivation (*GA2ox*) genes in seeds taken from red ripe fruit. Data are means± SE of 4 biological replicates. Statistical differences were determined by Student's t-test (P < 0.05); ns+ no significant difference.

Supplementary Tables

Supplementary Table S1. Primers used in this study.

Gene	Solyc ID number	Used for	Forward (5'-3')	Reverse (5'-3')
Actin	Solyc11g005330	RT-qPCR	GTCCTCTTCCAGCCATCCAT	ACCACTGAGCACAATGTTACCG
TINY1	Solyc06g066540	PCR	ATGTCAAAGCGAATAAGAGAGAGT	TTATTTCATCATTTCAAAGTTGCTAAG
TINY1	Solyc06g066540	RT-qPCR	TGGGTGTCTGAAATTCGCGA	TAGCCGCCATTTCTGCAGTT
GA20ox1	Solyc03g006880	RT-qPCR	AGATTGTGTTGGTGGACTTCAA	TAGCGCCATAAATGTGTCG
GA20ox2	Solyc06g035530	RT-qPCR	CGGTTTCTTTCTCGTGGCAA	TTTGCTTGTCGGAAAGTGGC
GA20ox3	Solyc11g072310	RT-qPCR	ACTTTAGGGACAGGGCCTCA	ACTTGAAGCCCACCAACACT
GA20ox4	Solyc01g093980	RT-qPCR	CAGCCACCACCCCTATTACA	ACGAGTGGCATTGGATACCG
GA3ox1	Solyc06g066820	RT-qPCR	AGTGTTTTACATCGGGCGGT	TCCACCAATTTCGACAGGGG
GA3ox2	Solyc03g119910	RT-qPCR	TTGGCCATGCATGCAAAACA	ATCTCGTCCCGTGTGTTTCC
GA3ox3	Solyc01g058250	RT-qPCR	TGGAACTTATGGGCCATGCA	AGTCGCCTTGCCTGAGATTC
GA3ox4	Solyc05g052740	RT-qPCR	CGCCTTGCCTGTAGAAGAGA	GAACCCTTCATGCCACATGA
GA3ox5	Solyc00g007180	RT-qPCR	TAGGCCATGCATGCAAAACA	GCTGGCAAGGCAAATAGTCG
GA2ox1	Solyc05g053340	RT-qPCR	AACTCTTGCAACTCCACGGT	ATTCGGGTGAGGTGATACGG
GA2ox2	Solyc07g056670	RT-qPCR	CATTCGGGCTGCGGTTAATG	TCCCTCCGCCAACATTTCAA
GA2ox4	Solyc07g061720	RT-qPCR	TTGAAAAGTTGGCGGAGGGA	AGCCTGAAAACAGAGTCGCT
GA2ox7	Solyc02g080120	RT-qPCR	AGCCACCTCCACTTCTCAAT	GGTTTGGCTGCTGTGACAAG
SEC3	Solyc07g025170	RT-qPCR	CAAGTTGTAATGTCGATCCGTGTT	AGCTTTTGTGGAGATTGCGAG
GID1b1	Solyc09g074270	RT-qPCR	GGCTGCTCTTCAATGGGTAA	TAAAACCTCGACGCCTGATT
TIP41	Solyc10g049850	RT-qPCR	ATCGAGTGTCGCAAGCTTTT	CGGCAAGTGAGTTGTCTGAA
35S:proΔ17	Solyc11g011260	PCR	CTATCCTTCGCAAGACCCTTCC	AAAACAGCACCACCAGGTAT
